# Extensive cutaneous involvement in pemphigus vulgaris in a pediatric patient: a rare clinical image

**DOI:** 10.11604/pamj.2026.53.19.49727

**Published:** 2026-01-16

**Authors:** Sahil Vihirkar, Darshana Kumari Wankhede

**Affiliations:** 1 Department of Child Health Nursing, Smt. Radhikabai Meghe Memorial College of Nursing Sawangi (Meghe), Datta Meghe Institute of Higher Education and Research, Sawangi (Meghe), Wardha, Maharashtra, India

**Keywords:** Pemphigus vulgaris, pediatrics, autoimmune blistering disease, skin

## Image in medicine

A 4-year-old child presented with multiple hyperpigmented and hypopigmented macules and plaques over both forearms, with a history of recurrent flaccid bullae and erosions at the same sites. Nikolsky sign was positive during the active phase. Tzanck smear showed acantholytic cells, and skin biopsy revealed intraepidermal acantholysis. Direct immunofluorescence demonstrated intercellular deposition of IgG and C3 in a fish-net pattern, confirming the diagnosis. The child was treated with systemic corticosteroids and azathioprine, leading to disease control. The present image shows the healing phase with post-inflammatory dyspigmentation and residual scarring, highlighting chronic sequelae of pediatric pemphigus vulgaris, a rare condition in this age group.

**Figure 1:**
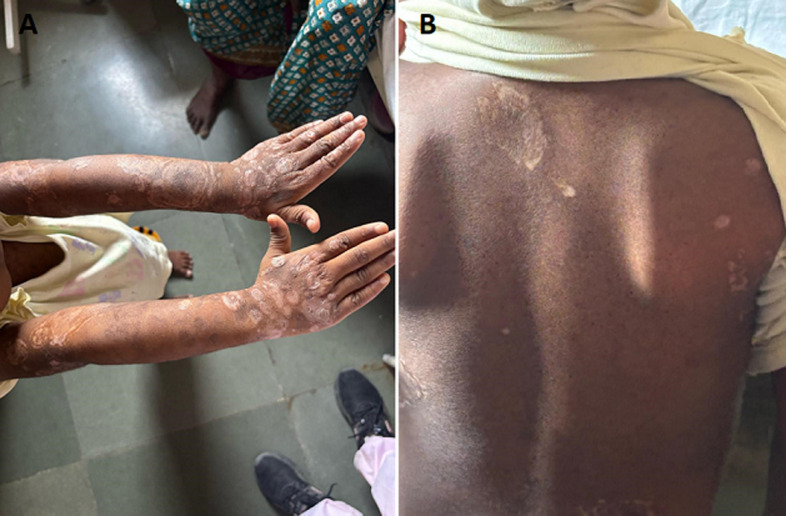
A) healed hyperpigmented and hypopigmented plaques over both forearms in a pediatric patient with a known history of pemphigus vulgaris; B) multiple hypopigmented and scarring lesions over the back in a child with previously treated tinea corporis (dermatophytosis)

